# An Approach to Improve the Quality of Infrared Images of Vein-Patterns

**DOI:** 10.3390/s111211447

**Published:** 2011-12-01

**Authors:** Chih-Lung Lin

**Affiliations:** 1 Department of Electronic Engineering, Hwa Hsia Institute of Technology, 111 Gong Jhuan Rd., Chung Ho district, New Taipei City, 23568, Taiwan; E-Mail: linclr@gmail.com; 2 Department of Information and Computer Engineering, Chung Yuan Christian University, 200 Chung Pei Rd., Chung Li City, 32023, Taiwan

**Keywords:** noise removal, noise detection, adaptive contrast enhancement, hybrid cumulative histogram equalization

## Abstract

This study develops an approach to improve the quality of infrared (IR) images of vein-patterns, which usually have noise, low contrast, low brightness and small objects of interest, thus requiring preprocessing to improve their quality. The main characteristics of the proposed approach are that no prior knowledge about the IR image is necessary and no parameters must be preset. Two main goals are sought: impulse noise reduction and adaptive contrast enhancement technologies. In our study, a fast median-based filter (FMBF) is developed as a noise reduction method. It is based on an IR imaging mechanism to detect the noisy pixels and on a modified median-based filter to remove the noisy pixels in IR images. FMBF has the advantage of a low computation load. In addition, FMBF can retain reasonably good edges and texture information when the size of the filter window increases. The most important advantage is that the peak signal-to-noise ratio (PSNR) caused by FMBF is higher than the PSNR caused by the median filter. A hybrid cumulative histogram equalization (HCHE) is proposed for adaptive contrast enhancement. HCHE can automatically generate a hybrid cumulative histogram (HCH) based on two different pieces of information about the image histogram. HCHE can improve the enhancement effect on hot objects rather than background. The experimental results are addressed and demonstrate that the proposed approach is feasible for use as an effective and adaptive process for enhancing the quality of IR vein-pattern images.

## 1. Introduction

Because the cost of IR cameras has declined in recent years, especially those that are not cooled, the number of applications based on IR images captured by un-cooled IR cameras has increased substantially. Many new applications of IR imaging have been developed and deployed in fields that include the military, medicine, industry, and biometric verification. Among the most important biometric verification applications is the capture of IR images of palm-veins [1–3] and faces [4,5]. However, IR images frequently have noise, low contrast, low brightness and small magnification of objects of interest, so that the images must be preprocessed to improve their quality for biometric verification.

However, the low signal-to-noise (S/N) ratio [6,7] is the inherent limitation of IR images that affects their quality and hinders their deployment. The low S/N ratio results in low signal and high noise that degrades the quality of IR images. This is significant for an un-cooled IR camera, which is much less expensive than a cooled camera and has been used more prevalently to capture IR images in recent years. The high noise is caused by the sensors and read-out circuits of IR cameras, and the low IR signal detected by IR sensors is caused by the degradation of the IR signal radiating from objects in bad atmospheric weather. To enhance image quality and improve the adoption of IR-based applications, some form of image preprocessing is necessary. Improvements in impulse noise removal and contrast enhancement are the crucial tasks of IR image preprocessing.

The standard median filter (MF) [8] has been prevalently used for noise removal in image preprocessing. However, the MF has an inherent limitation, which is its high computation load. The weighted median filter and the center-weight median filter (CWMF) [8,9] are modified to alleviate the inherent limitations of the MF at the expense of reduced noise removal performance. In addition, many methods [10–17] that combine the MF with impulse detection have been proposed to remedy the limitations of MFs. Their performance inherently relies on the performance of the impulse detector. Mean-based filters [18–20] are an alternative approach to remedy the limitations of the MF. These filters usually perform well at the cost of increased computational complexity. In recent years, some literature [21–24] has proposed impulse noise removal based on fuzzy technologies. However, all the previously mentioned literature considered visual images.

In this study, a FMBF to IR images based on an IR imaging mechanism to detect impulse noise and that is median-based to remove impulse noise with low computational load is developed. It can be performed without any prior knowledge about the IR image impulse noise, and no parameters must be preset.

In general, IR images consist of some objects of interest and many background areas. Not only is there a lack of prior information and much annoying noise, clutter, and stationary non-target objects [6,7], but it is also difficult to enhance the objects of interest without also enhancing the background or the noise. Using manual manipulation to enhance the gray value of objects of interest may be one way to remedy this problem, but it is not practical, because it is time-consuming and labor-intensive.

Many contrast enhancement technologies for IR images have been developed in the literature [25–29]. In [25], a self-adaptive contrast enhancement algorithm based on plateau histogram equalization was proposed, while [26] revealed that modeled histogram enhancement can considerably improve the poor detection performance associated with natural images. In [27] the authors used histogram equalization (HE) to develop plateau histogram equalization, and [28,29] used histogram projection. These technologies are all based on histogram information. In addition, some non-histogram-based enhancement techniques were developed in [30–35].

To enhance the IR images automatically, we have developed a novel adaptive contrast enhancement approach, which automatically generates a hybrid cumulative histogram (HCH) based on two different pieces of information about the image histogram. The proposed approach runs automatically, requiring no prior information about the IR images nor manually setting the parameters. This approach is called hybrid cumulative histogram equalization (HCHE). A reliable, robust and adaptive approach is based on FMBF, and HCHE is developed in this study.

**Figure 1.**
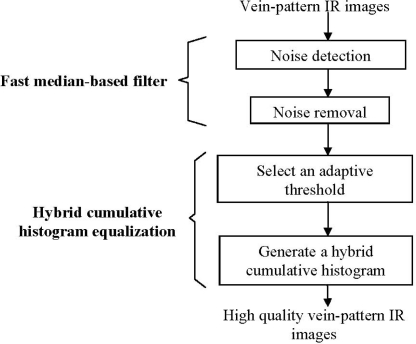
Demonstrates the block diagram of the proposed approach.

Figure 1 shows a block diagram of the proposed approach. It consists of four main parts, which are noise detection, noise removal, selecting an adaptive threshold and generating a hybrid cumulative histogram. They will be described in detail herein.

The rest of the paper is organized as follows. The FMBF approach with a lower computation load to reduce noise is addressed in Section 2. Section 3 describes the HCHE which generates a HCH for adaptive contrast enhancement to IR images. HCHE can remedy the shortcomings of the HE. Experimental results are demonstrated in Section 4 to verify the validity of the proposed approach, and concluding remarks are given in Section 5.

## 2. Fast Median-Based Filter (FMBF)

### 2.1. IR Imaging Mechanism

MF does not distinguish noisy pixels from regular pixels and processes every pixel in images, which results in increased computational load to process the signal pixels that do not need to be processed. Therefore, a good deal of computational load can be saved by performing noise reduction only on noise pixels. One of the ways to alleviate computing overload is to detect the noise pixels before performing any noise removal. The key point is how to detect noise pixels intelligently. To detect noise pixels in IR images, one must consider the imaging mechanism of IR images. The imaging mechanism is derived based on the heat radiation law and heat conduction law. The details have been described in [1].

According to the heat radiation law, the gray-level of pixels has a positive relationship with the temperature of the object surface. In addition, the heat conduction means that the temperature of the object varies monotonically on a surface. As a result, the crucial property of the *IR imaging mechanism* is that the gray-level of signal pixel of an object changes monotonically in IR images. On the contrary, the gray-level variations of noise pixels do not follow the *IR imaging mechanism*. FMBF is developed based on this crucial property of the *IR imaging mechanism* to detect noisy pixels.

### 2.2. Noise Detection

A number of impulse detection methods [11–17] have been proposed that combind with MF to reduce the impulse noise. They include several different types of methods. All of these methods detect impulse noise in visual images. In this study, a noise detection algorithm has been developed based on *IR imaging mechanism* to detect the noise in IR images.

In order to explain how to perform the noise detection, some parameters of image pixels must be defined first. The terms *x* and *y* represent the horizontal and vertical coordinates of a pixel, respectively. *p*(*x*,*y*) is the pixel with coordinates *x* and *y. g*(*x*,*y*) is the gray-level of the pixel *p*(*x*,*y*). *S_xy_* is the set that includes *g*(*x*,*y*) and its neighbor pixels. *gm* is the median gray-level of the *S_xy_*, *g*(*x*,*y*) represents the gray-level of the central position pixel that will be processed, (*s*,*t*) denotes the coordinates of the pixels belonging to *S_xy_*, and *g*(*s*,*t*) represents the gray-level of the pixels belonging to *S_xy_*.

According to the imaging mechanism of IR images, the gray-level of an object varies monotonically, which results in an important property: if the gray-level *g*(*x*,*y*) is equal to the maximum gray-level *g_max_* or the minimum gray-level *g_min_* inside a filter window, the central position pixel *p*(*x*,*y*) will be a noisy pixel and should be replaced. Otherwise, it is considered as a signal pixel and should not be changed. Hence, this paper proposes the noise detection algorithm to find noisy pixels inside the filter window in IR images based on two steps. First, the noise detection method employs Equations (1a) and (1b) to find the maximum and minimum gray-level inside the filter window. Next, the algorithm checks the gray-level *g(x*,*y*) with *g_min_* and *g_max_* to consider whether the pixel *p*(*x*,*y*) is noise or signal. This noise detection can be performed in the following steps.

Step 1. Determine the maximum and minimum gray-level inside the filter window.
  (1a) $$\begin{array}{l} g_{\max}=\mathrm{Arg}\text{max}\{g(s,t)\}\\ (s,t)\in S_{\mathrm{xy}} \end{array}$$
  (1b) $$\begin{array}{l} g_{\min}=\mathrm{Arg}\text{min}\{g(s,t)\}\\ (s,t)\in S_{\mathrm{xy}} \end{array}$$
Step 2. Based on the IR imaging mechanism, check *g*(*x*,*y*) with gray-level *g_min_* and *g_max_* to determine whether the pixel *p*(*x*,*y*) is noise or signal.
  (2) $$\left\{ \begin{array}{l} \mathrm{If}\;g(x,y)=g_{\min}\;\text{or}\;g_{\max}p(x,y)\;\text{is a noise}\\ \text{Otherwise}\;p(x,y)\;\text{is considered as a signal pixel} \end{array}\right.$$

The proposed noise detection is based on the *IR imaging mechanism*. Therefore, FMBF can exactly identify the noisy pixels and replace them in IR images. The IR image’s edges, text information and details of objects are not damaged while being processed by the proposed FMBF.

### 2.3. Noise Removal

According to the property of *IR imaging mechanism*, the pixel with median gray-level inside the window is adopted replacing the noisy pixels. In the proposed approach, the procedure to find out median gray-level is performed by the sort algorithm with a low computation complexity. In addition, it only processes the noisy pixels, but not the signal pixels.

Sorting is the main computation load of the noise removal, so in order to speed up the noise removal, reducing the computational load is critical. This study adopts a sort algorithm with low computational load. From an analysis of the properties of the versatile sort algorithms, a Radix sort with suitable parameters should run faster even than Quick sort or Heap sort [36]. The complexity of Radix sort is described as Equation (3):

(3) $$\text{Complexity of Radix sort}\;=\;\text{O}\left(n\;{\text{log}}_{d}\;q\right)$$

where *n* is the number of data, *d* is the number of digits, *q* is the range of the digit.

Thus in the proposed approach, a Radix sort algorithm with suitable *d* and *q* is selected to sort the pixel gray-level. In order to reduce the computational load of the Radix sort algorithm further, a Bit-plane processing technique is utilized to perform the Radix sort algorithm. The Bit-plane processing is easily implemented by using hardware in parallelization [37]. It can be achieved by the Bit-plane decomposition method [38], which decomposes the IR image into *m* bit-planes, where *m* is the number of bits in each pixel. Then the Radix sort algorithm is performed on these bit-planes to select the pixel with the median gray-level *g_m_* inside a filter window. The selected pixel with gray-level *g_m_* is utilized to replace the noisy pixel in the central position inside the filter window and the noise removal is accomplished.

FMBF does not act like the MF in sorting each pixel in a whole IR image; it only processes noisy pixels. In general, there are far fewer noisy pixels than signal pixels. FMBF has to expend extra computational load to determine the maximum and minimum gray-level in the noise detection procedure. The computation complexity of doing is O(*n*), which is still less than the computational complexity of Radix sort O(*n* log*_d_* *q*). Thus, the proposed FMBF can theoretically save time in noise removal.

## 3. Hybrid Cumulative Histogram Equalization (HCHE)

Histogram equalization (HE) is the most popular method utilized to enhance images, but it has a limitation in that it enhances primarily the large area scene features with approximate gray-level, rather than the small area objects [25]. In our work, a HCHE is developed to adaptively and effectively enhance IR images. The most important property of HCHE is that the enhancement effect on hot objects is more than that on large area backgrounds. HCHE consists of two stages: the adaptive threshold selection and the HCH generation. The first stage adaptively selects a suitable threshold that divides the histogram into hot objects and backgrounds. The second stage is based on two different kinds of information about the histogram to generate two different cumulative histograms. One enhances hot objects and the other enhances backgrounds. Then the two different cumulative histograms are combined into a HCH. Thus, HCHE can remedy the inherent limitation of HE. The details of how to perform HCHE are addressed in the following section.

### 3.1. Selecting an Adaptive Threshold

In order to automatically divide the histogram into hot objects and backgrounds for miscellaneous IR images, an adaptive threshold is needed. Abundant threshold selection methods have been described in [39] and have been categorized according to their information, such as entropy, histogram shape, spatial correlation, measurement space clustering, object attributes, and local gray-level surface. A fixed threshold is unsuitable to address the variations in different IR images. In order to conquer this real problem, this paper applies the iterative threshold selection method [40], based on the clustering-based thresholding method, to choose an adaptive threshold. The iterative threshold selection method has been effective in extracting adaptive thresholds from bimoded or non-bimoded histograms [1,41]. The procedures of the iterative threshold selection method are as follows:

Step 1. Initialize the threshold *Th*(*1*) (0 < *Th*(*1*) < 255) with a random value to segment the image into background and object.
Step 2. At the k^th^ iteration, compute *μ_B_(k)* and *μ_o_(k)* as the means of background and object gray-level, respectively. The threshold *Th*(*k*) adopted in segmenting images into the background and the object is determined in Step 3 of the previous iteration (Equation (6)).
  (4) $$\mu_{B}=\sum_{(i,j)\in \mathrm{background}}f(i,j)/N_{B}$$
  (5) $$\mu_{O}=\sum_{(i,j)\in \mathrm{object}}f(i,j)/N_{O}$$
  where *N_B_* and *N_O_* are the pixel numbers of the background and the object, respectively.
Step 3.
  (6) $$\text{Set}\;\mathrm{Th}(k+1)=\left(\mu_{B}(k)+\mu_{0}(k)\right)/2$$
  *Th*(*k* + *1*) is the new threshold for the next iteration.
Step 4. If *Th*(*k* + *1*) = *Th*(*k*), then terminate and name the selected threshold *Th*; otherwise return to Step 2.

### 3.2. Generating a Hybrid Cumulative Histogram

The enhanced effects caused by HE will be less obvious on hot objects than on large area backgrounds. In order to remedy this shortcoming of HE, this paper proposes a HCHE, whose main property is the use of two different enhancement effects on hot objects and backgrounds: a large effect on hot objects and a small effect on backgrounds. The details for constructing a HCH follow.

In order to explain the generation of HCH, the cumulative histogram of HE, Equation (7), and its associated parameters are defined first:

(7) $$T\left(r_{k}\right)=\sum_{j=0}^{k}p_{r}\left(r_{j}\right)=\sum_{j=0}^{k}n_{j}/n\;\;\;\;\;\;\;\;k=0,\;\;1,\;\;2,\ldots ,\;L-1$$

(8) $$p_{r}\left(r_{k}\right)=n_{k}/n\;\;\;\;\;k=0,\;\;1,\;\;2,\ldots ,\;L-1$$

(9) $$s_{k}=\text{int}\left(\left(L-1\right)\times T\left(r_{k}\right)\right)$$

where *r_k_* is the gray-level in the histogram of the source image, *k* is the gray-level, *n* is the total number of pixels in an image, *n_k_* is the number of pixels with gray-level *r_k_*, *p_r_*(*r_k_*) is the probability of the pixel with gray-level *r_k_*, *s_k_* is the pixel gray-level in the histogram of processed images, *L* is the number of gray-levels, *L* = 256 in our case, *T*(*r_k_*) is the cumulative histogram of the HE generated by the source image, and int() is a function giving the integral part of a decimal number.

Based on the selected threshold *Th*, the histogram is divided into two groups: the pixels with gray-level smaller than *Th*, which belong to the background, and the pixels with gray-level equal to or larger than *Th*, which belong to the hot objects. The cumulative histogram to enhance the background is constructed by using the probability density function (PDF) of the background histogram, which is expressed in Equation (10a). The intensity density function (IDF) is defined as Equation (10c). The cumulative histogram to enhance hot objects is generated by using the IDF of the hot object histogram, which is expressed in Equation (10b). The HCH is constructed by combining Equations (10a) and (10b):

(10a) $$T_{\mathrm{hb}}\left(r_{k}\right)=\{\sum_{j=0}^{k}\mathrm{Arg}\;\text{min}\left\{p_{r}\left(r_{j}\right),p_{r}\left(r_{\mathrm{Th}}\right)\right\}\;\;\;\;\;\;\;\;\;\;\;\;0\leq k<\mathrm{Th}$$

(10b) $$T_{\mathrm{hb}}\left(r_{k}\right)=\{\sum_{j=\mathrm{Th}}^{k}p_{r}\left(r_{j}\right)\times {\text{log}}_{2}j\;\;\;\;\;\;\;\;\;\;\;\;\;\;\;\;\;\;\;\;\;\;\;\;\mathrm{Th}\leq k\leq L-1$$

(10c) $$\text{IDF}\;=\;p_{r}\left(r_{k}\right)\times {\text{log}}_{2}k0\leq k\leq L-1$$

where *T_hb_*(*r_k_*) is the HCH.

Equation (10a) accumulates the minimum of probability *p_r_*(*r_j_*) and *p_r_*(*r_Th_*) to generate the cumulative histogram for enhancing the background. Equation (10b) accumulates the IDF of probability *p_r_*(*r_j_*) to generate the cumulative histogram for enhancing hot objects. Combining Equations (10a) and (10b) generates the HCH of HCHE, which possesses two different enhancement effects on backgrounds and hot objects.

The derivative of a function is defined as the ratio of Δ*y* and Δ*x*. The terms Δ*y* and Δ*x* represent the argument increment of a function in Y-axis and X-axis, respectively. In general, the argument in Y-axis is the output and the argument in X-axis is the input of a function. Thus, the derivative of cumulative histogram is large which reveals the enhancement effect of cumulative histogram is large. Similarly, the derivative of cumulative histogram is small which reveals the enhancement effect of cumulative histogram is small. Therefore, the derivative of cumulative histogram can represent the enhancement effect of cumulative histogram.

Comparing Equations (7) with (10a), it is clear that the derivative of Equation (10a) is less than or equal to the derivative of the cumulative histogram of the HE when the gray-level *r_k_* belongs to the background. Then Equation (10b) constructs the cumulative histogram of the hot objects. Comparing Equations (7) with (10b), it is easy to observe that the derivative of Equation (10b) must be larger than or equal to the derivative of Equation (7) when the gray-level *r_k_* represents the hot objects. Thus, the hot objects’ enhanced effect caused by the HCH is larger, and the backgrounds’ is smaller. This result can be proven based on the *backward difference approximation of the first derivative* (BDAFD) [42]. According to the BDAFD, the first derivative of a cumulative histogram of the HE can be expressed as:

(11) $$\begin{array}{l} T'\left(r_{k}\right)=\left(T\left(r_{k}\right)-T\left(r_{k-1}\right)\right)/\left(r_{k}-r_{k-1}\right)\\ \;\;\;\;\;\;\;\;\;\;\;\;\;=\left(\sum_{j=0}^{k}p_{r}(r_{j})-\sum_{j=1}^{k-1}p_{r}(r_{j})\right)/1\\ \;\;\;\;\;\;\;\;\;\;\;\;\;=p_{r}\left(r_{k}\right) \end{array}$$

where *T*’(*r_k_*) is a first derivative of the cumulative histogram *T*(*r_k_*).

In the same manner, the first derivative of the HCH can be expressed as:

(12) Thb'(rk)=(Thb(rk)−Thb(rk−1))/(rk−rk−1)     ={Arg min {pr(rk),pr(rTh)}0≤k<Thpr(rk)×log2rkTh≤k<L−1

where *T_hb_*’(*r_k_*) is a first derivative of the HCH *T_hb_*(*r_k_*). If gray-level *r_k_* belongs to the background, *i.e.*, 0 ≤ *k* < *Th*, it yields:

$$\mathrm{Arg}\;\text{min}\left\{p_{r}\left(r_{k}\right),p_{r}\left(r_{\mathrm{Th}}\right)\right\}\leq p_{r}\left(r_{k}\right)$$

So:

(13) Thb'(rk)≤T'(rk)                            0≤k<Th

Thus, the background’s enhancement effect caused by the HCH Equation (10a) is smaller than or equal to that caused by the cumulative histogram of the HE. When the gray-level *r_k_* belongs to the object, *i.e*., *Th* ≤ *k* < *L* − 1, it yields:

$$p_{r}\left(r_{k}\right)\times {\text{log}}_{2}r_{k}\geq p_{r}\left(r_{k}\right)$$

Thus:

(14) Thb'(rk)≥T'(rk)                            Th≤k<L−1

meaning that the enhancement effect on objects caused by the HCH, Equation (10b) is larger than or equal to that caused by the cumulative histogram of the HE.

As a result, the enhancement effect on hot objects caused by HCHE is larger than or equal to that caused by the HE. On the other hand, the enhancement effect on backgrounds caused by HCHE is smaller than or equal to that caused by the HE. This characteristic is especially significant when the hot objects are much smaller than the backgrounds. Consequently, HCHE proposed in this study can alleviate the limitation of HE.

In addition, in order to avoid saturation of the equalized gray-level of transformed pixels, the maximum gray-level of transformed pixels must remain less than or equal to the maximum scale of the gray-level. A normalized version of HCHE is needed and is expressed as:

(15) $$T_{n}\left(r_{k}\right)=T_{\mathrm{hb}}\left(r_{k}\right)/T_{\mathrm{hb}}\left(r_{L-1}\right)\;\;\;\;\;\;\;\;\;\;\;\;\;\;\;\;\;\;\;\;\;\;\;\;\;\;\;\;0≦k≦L-1$$

where *T_n_*(*r_k_*) is the normalization of *T_hb_*(*r_k_*).

## 4. Experiment

A typical noise measure used is peak signal-to-noise ratio (PSNR) [8], defined as:

(16) $${\text{PSNR}\;=\;10\;\text{log}}_{10}\left({\text{Max}}^{2}/\text{MSE}\right)$$

where MSE is the mean square error between the original and processed image and Max is the maximum gray scale of pixels, e.g., 255 for 8 bits.

### 4.1. Test Samples of the Vein-Pattern IR Images

**Figure 2.**
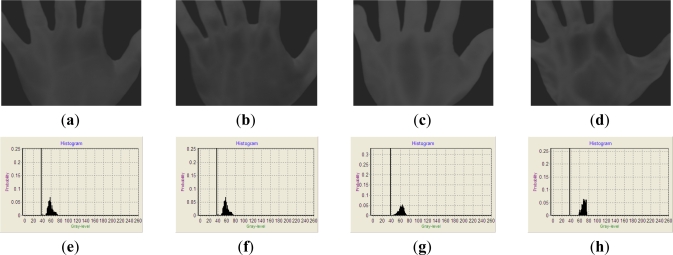
Images (**a**–**d**) demonstrate the thermal images captured from four different palm-dorsa. Images (**e**–**h**) show the corresponding histograms of (a–d).

In order to verify the validity of the proposed approach, four life-time IR images of palm-dorsa are collected and used as the test samples for our study. Each IR image has 640 H × 480 V pixels and each pixel is represented by 256 gray-levels. As shown in Figure 2, the IR images all display low contrast and brightness. The vein-patterns are hard to observe in these images. They have to be preprocessed to improve the image quality for the future postprocessing.

### 4.2. Experimental Results

**Figure 3.**
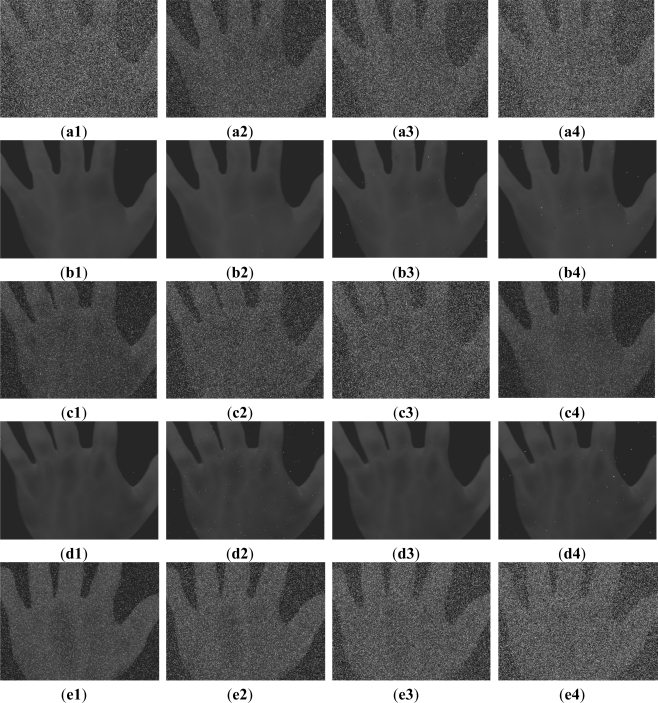

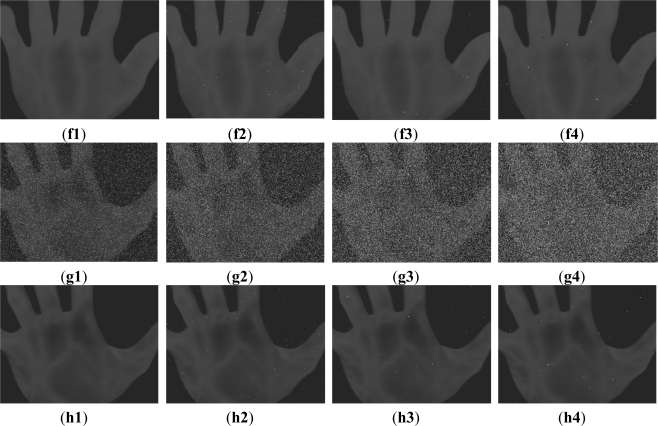
Figure 2(a–d) with impulse noise filtered by FMBF. (**a1**–**a4**) show Figure 2(a) with impulse noise of 10%, 20%, 30% and 40%, respectively; (**b1**–**b4**) show the results of (a1–a4) filtered by FMBF once, once, two times and three times, respectively; (**c1**–**c4**) show Figure 2(b) with impulse noise of 10%, 20%, 30% and 40%, respectively; (**d1**–**d4**) show the results of (c1–c4) filtered by FMBF once, once, two times and three times, respectively; (**e1**–**e4**) show Figure 2(c) with impulse noise of 10%, 20%, 30% and 40%, respectively; (**f1**–**f4**) show the results of (e1–e4) filtered by FMBF once, once, two times and three times, respectively; (**g1**–**g4**) show Figure 2(d) with impulse noise of 10%, 20%, 30% and 40%, respectively; (**h1**–**h4**) show the results of (g1–g4) filtered by FMBF once, once, two times and three times, respectively.

Figure 3 shows the Figure 2(a–d) with impulse noise of 10%, 20%, 30% and 40%, and exhibit the results of performing FMBF to iteratively filter the noisy images. Considering Figure 3(a1–a4) as examples, Figure 3(a1) is filtered once, Figure 3(a2) filtered once, Figure 3(a3) is iteratively filtered two times, Figure 3(a4) is iteratively filtered three times, respectively. Figure 3(b1,b2) demonstrate how the impulse noise is filtered out and the edges, textures and detail information preserved simultaneously. Figure 3(b3,b4) demonstrate how the impulse noise is almost filtered out except for a few noise with large area. The other filtered images as shown in Figure 3 are quite consistent with the Figure 3(b1–b4).

The PSNR is applied to assess the noise reduction performance of FMBF. The PSNRs of Figure 3(a–d) caused by FMBF are addressed in Table 1. Using Figure 3(a1) as an example, the PSNR caused by FMBF is 57.90 dB, meaning that the degree of noise reduced by FMBF is 20.82 dB. It is easy to observe, the noise in Figure 3(b1) is less than in Figure 3(a1), which shows the consistence with the PSNR. Table I also shows the PSNRs caused by FMBF of Figure 3(b2–b4) are 51.32 dB, 53.28 dB and 52.60 dB, respectively. This shows that FMBF reduces noise by 17.26 dB, 20.98 dB and 21.52 dB for Figure 3(b2–b4), respectively. Compared to the MF, FMBF improves the PSNRs 2.67 dB, 1.03 dB, 1.44 dB and 2.03dB for Figure 3(a1–a4), respectively.

Table 1 also illustrates the sort time performance of applying FMBF to noisy images shown as Figure 3(a1–a4), (c1–c4), (e1–e4) and (g1–g4). FMBF performs a sorting algorithm for noisy pixels only, performing the sorting algorithm 204,500 times for Figure 3(a1). Hence, the computation load is reduced by the proposed FMBF by 33.4%. FMBF reduces the computation load 44.1%, 32.1% and 25.2% for Figure 3(a2–a4), respectively. These experimental results are quite consistent with the aforementioned theoretical analysis.

In the same manner, Table 1 also illustrates the results of Figure 3(a1–a4) filtered by FMBF. All the Figure 3 and Table 1 show that the performances of the proposed FMBF can reduce impulse noise and computer load more effectively than MF. In addition, FMBF performs the Radix sort algorithm with bit-plane, so it is easily implemented by hardware in parallelization. Thus, FMBF can save further computational time when performed in a parallel hardware architecture.

**Figure 4.**
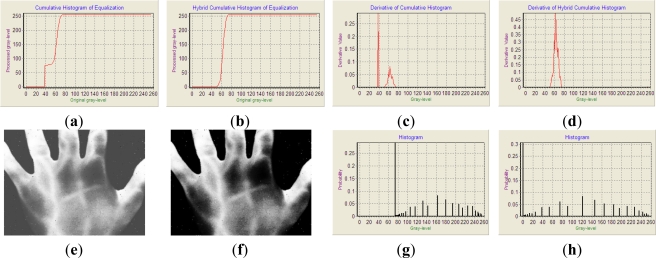
The experimental results of Figure 3(b2) enhanced by the HE and HCHE. (**a**) A normalized cumulative histogram of the HE. Y-axis and X-axis represent processed gray-level and original gray-level, respectively; (**b**) The normalized HCH of the HCHE with the adaptive threshold 51. Y-axis and X-axis represent processed gray-level and original gray-level, respectively; (**c**) The derivative of the cumulative histogram. Y-axis and X-axis represent derivative value and gray-level, respectively; (**d**) The derivative of the HCH. Y-axis and X-axis represent derivative value and gray-level, respectively; (**e**) The Figure 3(b2) enhanced by the HE; (**f**) The Figure 3(b2) enhanced by the HCHE; (**g**) The histogram of (e). Y-axis and X-axis represent probability and gray-level, respectively; (**h**) The histogram of (f). Y-axis and X-axis represent probability and gray-level, respectively.

Figure 4 demonstrates the experimental results of Figure 3(b2) enhanced by the HE and HCHE with a threshold of 51. The image enhanced by HE is shown in Figure 4(e), and the histogram of Figure 4(e) is shown as Figure 4(g). Figure 4(e,g) reveal that the intensities of the backgrounds are increased, and the contrast is spread. Intensities of the hot objects, vein-patterns, are also increased to near-maximum gray scale, but their contrast is compressed to a narrow gray-level range. On the other hand, when one observes the derivative of the HCH of the HCHE, as shown in Figure 4(d), one can see that the derivative in the interval from gray-level 37 to 51 is less than that in the interval from gray-level 51 to 74. The former represents gray-levels belonging to backgrounds, and the latter represents gray-levels belonging to hot objects. Thus, the enhancement effect on backgrounds induced by HCHE is less than that on hot objects, and the contrast of backgrounds is decreased, whereas the contrast of hot objects is increased. The enhanced image by the HCHE is addressed in Figure 4(f), and the histogram of Figure 4(f) is shown as Figure 4(h). The gray-levels of backgrounds in Figure 4(h) are decreased and the contrast is compressed, while the gray-levels of hot objects are increased and the contrast is spread. These figures show that the experimental results in the cumulative histogram and the HCH match well with Equations (8,9) and the proposed HCHE can remedy the limitation of the HE.

**Figure 5.**
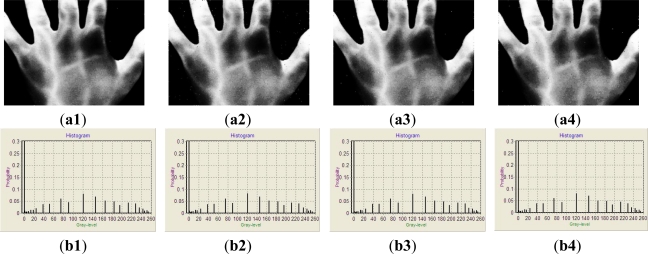

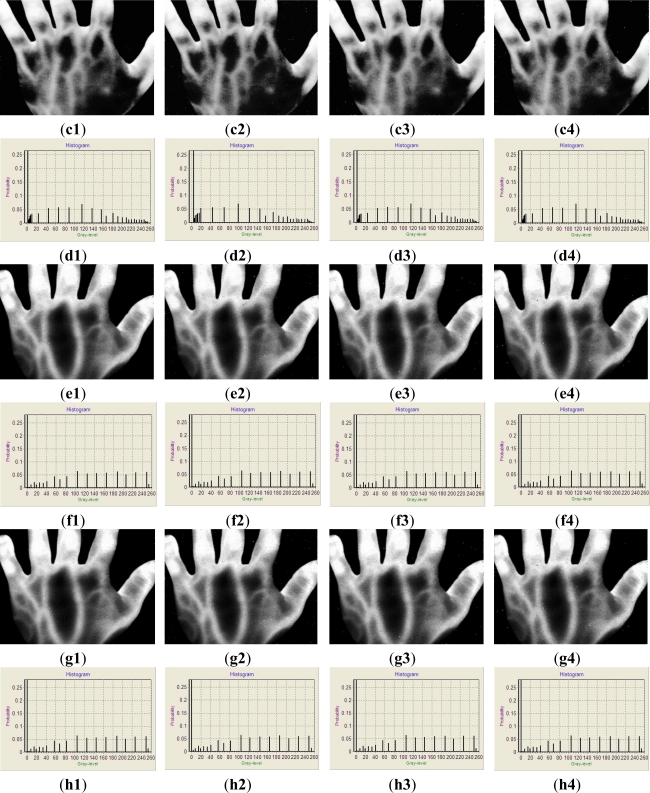
The results enhanced by HCHE. (**a1**–**a4**) show the results of Figure 3(b1–b4) enhanced by HCHE, respectively; (**b1**–**b4**) show the histograms of (a1–a4), respectively; (**c1**–**c4**) show the results of Figure 3(d1–d4) enhanced by HCHE, respectively; (**d1**–**d4**) show the histograms of (c1–c4), respectively; (**e1**–**e4**) show the results of Figure 3(f1–f4) enhanced by HCHE, respectively; (**f1**–**f4**) show the histograms of (e1–e4), respectively; (**g1**–**g4**) show the results of Figure 3(h1–h4) enhanced by HCHE, respectively; (**h1**–**h4**) show the histograms of (g1–g4), respectively.

Figure 5 demonstrates the performance of HCHE. The results of Figure 3(b1–b4) enhanced by HCHE are shown as Figure 5(a1–a4). Figure 5(b1–b4) show the histograms of Figure 5(a1–a4). Compared to Figure 3(b1–b4), the contrast of images enhanced by HCHE is better than original images and the vein-patterns are much clearer and easier to observe. Figure 5 exhibits the high performance of HCHE to enhance vein-patterns IR images.

## 5. Conclusions

This paper presents an approach to improve the quality of IR images of vein-patterns by using the FMBF and HCHE with HCH. The proposed approach has three main advantages. The first is that noise reduction is achieved by utilizing FMBF with noise detection, and noise removal is performed by Radix sorting with bit-plane decomposition, which effectively decreases the computational load for noise reduction. In addition, FMBF speeds up the computation while preserving the benefits and remedying the shortcomings of the median filter. The second advantage of this approach is that HCHE with HCH, which is based on using information about the histogram to enhance IR images, has a greater enhancement effect on hot objects than on large backgrounds. This property can remedy the limitation of the HE. Finally, no prior knowledge about the IR images is necessary, and no parameter must be manually preset to perform the proposed approach.

IR images often display low intensity, low contrast and high noise. It is a considerable challenge to provide a high enhancement effect on hot objects but not on backgrounds and to simultaneously reduce the noise. To overcome this challenge, this paper proposes an approach consisting of the FMBF and HCHE. Experimental results demonstrate that the proposed approach successfully meets this challenge and extends the IR image applications in the military, medicine, industry and biometric verification fields.

## Figures and Tables

**Table 1.** The improved performances of the images processed by FMBF.

**Noise**			**10%**	**20%**	**30%**	**40%**

	**Filtered time**		**1**	**1**	**2**	**3**
Figure 2(a)	PSNR (dB)	Noisy image	37.08	34.06	32.30	31.08

		Median Filter	55.23	50.29	51.84	50.57
		FMBF	57.90	51.32	53.28	52.60
		Improved (db)	2.67	1.03	1.44	2.03

	Sort times	FMBF	204,500	171,813	416,922	689,883
		Improved (%) *	33.4	44.1	32.1	25.2
Figure 2(b)	PSNR (dB)	Noisy image	37.03	34.10	32.28	31.06
		Median Filter	55.29	51.60	52.22	50.97
		FMBF	57.74	53.37	53.84	53.26
		Improved (db)	2.45	1.77	1.62	2.29
	Sort times	FMBF	197,990	166,899	406,958	674,561
		Improved (%) *	35.6	45.7	33.8	26.8

Figure 2(c)	PSNR (dB)	Noisy image	37.07	34.02	32.28	31.04
		Median Filter	54.73	50.56	51.91	50.24
		FMBF	57.55	52.47	53.39	52.11
		Improved (db)	2.82	1.91	1.48	1.87
	Sort times	FMBF	207,747	173,809	420,298	693,919
		Improved (%) *	32.4	43.4	31.6	24.7

Figure 2(d)	PSNR (dB)	Noisy image	37.08	34.10	32.28	31.08
		Median Filter	55.01	51.34	52.27	51.19
		FMBF	57.74	53.21	53.78	53.07
		Improved (db)	2.73	1.87	1.51	1.88
	Sort times	FMBF	203,735	172,463	408,414	673,432
		Improved (%) *	33.7	43.9	33.5	26.9

* Compared to median filter sorts whole image which needs 307,200 times per filter time.
